# Can Unsupervised Machine Learning Support Malnutrition Risk Screening in Older Adults? A Preliminary Study Using Multidimensional Phenotyping and Hierarchical Clustering on Principal Components

**DOI:** 10.3390/nu18152560

**Published:** 2026-08-05

**Authors:** Karolina Kujawowicz, Iwona Mirończuk-Chodakowska, Monika Cyuńczyk, Anna Maria Witkowska

**Affiliations:** Department of Food Biotechnology, Medical University of Białystok, ul. Szpitalna 37, 15-285 Białystok, Poland; iwona.mironczuk-chodakowska@umb.edu.pl (I.M.-C.); monika.cyunczyk@umb.edu.pl (M.C.); anna.witkowska@umb.edu.pl (A.M.W.)

**Keywords:** malnutrition, hierarchical clustering on principal components, older adults

## Abstract

**Background/Objectives**: Malnutrition in older adults constitutes a significant public health concern, reflecting a multifactorial risk and demonstrating the necessity of early identification in geriatric care. This preliminary study aimed to explore whether an unsupervised machine learning (ML) approach based on hierarchical clustering on principal components (HCPC) could help characterize multidimensional phenotypes associated with malnutrition risk in older adults. **Methods**: This exploratory cross-sectional study included 105 older adults, aged 60–95 years, recruited from community and residential care settings. Anthropometric measurements, body composition, nutritional status, depressive symptoms, physical and functional fitness, frailty, sarcopenia, appetite, and biological and hematological parameters were evaluated. The statistical analyses were performed using ML combining Multiple Factor Analysis (MFA) for dimensionality reduction and HCPC for cluster identification. **Results**: HCPC identified three distinct phenotypes. Cluster 1 represented a relatively preserved nutritional and geriatric phenotype with lower frailty, residential care, and disease burden. Cluster 2 was a male-dominant group with preserved muscle mass and functional reserve. Cluster 3 was the most vulnerable phenotype, characterized by higher malnutrition risk, frailty, polypharmacy, depressive symptoms, and poorer functional status, with the strongest links observed for body composition, muscle mass, grip strength, malnutrition risk, frailty risk, and functional performance. **Conclusions**: This preliminary study suggests that unsupervised machine learning may help characterize geriatric and nutritional profiles associated with malnutrition risk in older adults. Higher malnutrition risk was observed in the profile characterized by greater multidimensional vulnerability, warranting cautious interpretation and validation in larger cohorts.

## 1. Introduction

Malnutrition and the risk of malnutrition in older adults represent a major public health concern, as they are associated with increased morbidity, mortality, functional decline, institutionalization and healthcare costs [1]. Reported prevalence rates vary widely depending on the population studied, clinical setting, and screening tool used, ranging from a few percent to several tens of percent [2,3]. The highest burden is generally observed among older adults in hospitals, rehabilitation facilities, and long-term care institutions, where multimorbidity, functional impairment and frailty frequently coexist [4].

Older adults are particularly vulnerable to nutritional disturbances due to age-related physiological changes, chronic diseases, polypharmacy, functional limitations, and socioeconomic factors [5,6]. Sarcopenia, reduced appetite, depressive symptoms, loneliness, and limited access to nutritious food may further increase the risk of malnutrition and frailty [7,8,9]. As malnutrition contributes to impaired immune function, delayed recovery, reduced physical performance, falls, prolonged hospitalization, and institutionalization, early identification of individuals at risk remains an important clinical and public health priority [10,11].

The Mini Nutritional Assessment (MNA) is widely used to identify older adults who are well nourished, at risk of malnutrition or malnourished [12]. However, nutritional status in geriatric patients is shaped by multiple interacting clinical, functional, biochemical, and psychosocial factors [13,14,15]. Therefore, MNA-based classification may be strengthened by a broader multidimensional characterization of patients, allowing the identification of clinically meaningful profiles of malnutrition risk [16,17,18].

Data-driven methods, including principal component analysis (PCA) and cluster analysis, provide a methodological framework for exploring complex multidimensional patterns in geriatric populations [19]. Hierarchical clustering on principal components (HCPC) combines dimensionality reduction with cluster analysis and enables the identification of relatively homogeneous subgroups within a multidimensional clinical space [20]. This approach is relevant to the assessment of nutritional status, as older adults with similar nutritional screening results may differ substantially in functional capacity, comorbidity burden, biochemical profile, frailty, and overall vulnerability [21].

Therefore, the aim of this study was to preliminarily assess whether hierarchical clustering on principal components (HCPC) can help characterize multidimensional profiles of older adults associated with malnutrition risk. The analysis combined MNA-based nutritional assessment with anthropometric, functional, clinical, and biochemical variables collected as part of comprehensive geriatric assessment. To our knowledge, this is one of the first studies to apply HCPC to multidimensional CGA-derived variables, including functional, clinical, and laboratory measures, to characterize profiles associated with malnutrition risk defined by the Mini Nutritional Assessment (MNA) in older adults.

## 2. Materials and Methods

### 2.1. Study Group

The cross-sectional study included 105 older adults aged 60–95 years (79.0% women; mean age 70.0 ± 6.4 years). Eighty-one participants were recruited from the community and 24 from residential care settings. The study used a consecutively recruited convenience sample and the analysis was exploratory. Recruitment may have varied by sex, living arrangement, and age group because of differences in availability, willingness to participate and functional status; this was considered when interpreting the sample.

#### Characteristics of the Studied Sample

Table 1 presents the baseline characteristics of the 105 older adults included in the study. The table includes demographic variables, such as age, sex, education level, and residence; the prevalence of major comorbidities; medication use, including polypharmacy and selected drug classes; nutritional and functional risk status, including malnutrition assessed using the MNA, CNAQ, and SNAQ, frailty, and sarcopenia identified using the SARC-F; anthropometric parameters, including BMI and body fat percentage; and selected laboratory markers, including albumin, prealbumin, CRP, total cholesterol, LDL, and HDL. According to the MNA classification, all participants were categorized as at risk of malnutrition and none met the criterion for malnutrition. Accordingly, the MNA assessment is reported as the primary outcome, with participants described as either without malnutrition risk or at risk of malnutrition. Hypothyroidism and hyperthyroidism were analyzed as separate indicators, while the hyperthyroidism variable is noted in Appendix A as requiring verification.

### 2.2. Participant Recruitment Process

**Figure 1:**
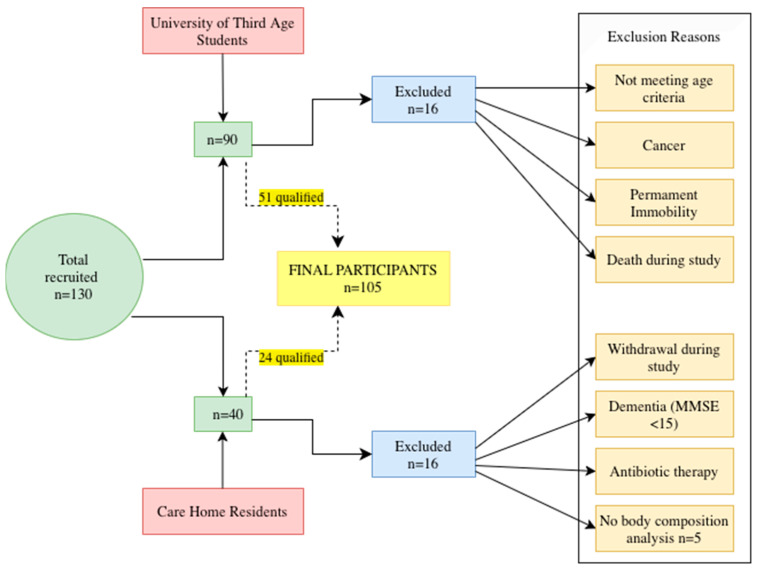
Flowchart of participant recruitment process.

Initially, 130 individuals were recruited for the study. After verification of the inclusion and exclusion criteria, 105 participants were ultimately enrolled. The inclusion criteria were age ≥ 60 years, willingness to participate in the study, BMI ≥ 18.5 kg/m^2^, ability to walk without assistance, stable chronic conditions, absence of fever or active infection, no current antibiotic therapy, no parenteral or enteral nutrition, no severe liver or kidney failure within the preceding 30 days, no active cancer within the previous 5 years, and serum samples meeting the laboratory requirements. All participants followed a standard diet. Twenty-five individuals were excluded for the following reasons: age < 60 years, cancer, permanent immobilization, death, withdrawal from the study, dementia defined as MMSE < 15, or lack of body composition analysis. The study included a restricted population of ambulatory, clinically stable older adults. The recruitment flowchart is presented in Figure 1.

The study was conducted in accordance with the principles of the Declaration of Helsinki and applicable ethical standards, with the aim of protecting the autonomy, privacy, and dignity of the participants. The implemented procedures were designed to achieve two primary objectives: first, to obtain reliable results, and second, to ensure the safety and comfort of all individuals involved. The study was approved by the Bioethics Committee of the Medical University of Białystok (approval no. APK.002.421.2021).

### 2.3. Data Collection

The data were collected through a structured interview conducted by a trained research team with appropriate qualifications and experience in geriatric assessment. Clinical information included a detailed medical history, the number of medications currently taken, and details of comorbidities, enabling a detailed characterization of participants’ health status. A comprehensive geriatric assessment was performed to evaluate the mental, physical, functional, clinical and anthropometric status of the study participants.

The dataset included demographic and clinical variables, including age, sex, education, medication intake, height, body weight, body mass index (BMI), waist-to-hip ratio (WHR), upper arm circumference (MAC), and right calf circumference (CC). Particular attention was given to polypharmacy, defined as the routine use of at least five medications per day [22]. The composition of the treatment regimen was also examined, including analgesics, statins, antidiabetic agents, neurological agents, anti-inflammatory agents, anticoagulants, antihypertensives, antidepressants, medications used for hypothyroidism, and diuretics. Data on comorbidities included hypertension, diabetes mellitus (DM), gastroesophageal reflux disease (GERD), heart disease, hypothyroidism and hyperthyroidism and degenerative diseases of the spine and joints. Information was also collected on serious illnesses and hospitalizations during the preceding year, smoking status, alcohol consumption and meal regularity. All physical measurements were obtained during a single morning visit, between 8:00 and 10:00 a.m., under fasting, resting conditions.

#### 2.3.1. Anthropometric Parameters and Body Composition Analysis

Body composition was assessed using the InBody 270 body composition analyzer (Biospace, Los Angeles, CA, USA) [23]. The following parameters were evaluated: body weight, BMI (kg/m^2^), WHR, and visceral fat level (VFL). Additionally, total body water (TBW, L), protein mass (kg), mineral mass (kg), fat-free mass (FFM, kg), body fat mass (BFM, kg), skeletal muscle mass (SMM, kg), percentage body fat (PBF, %) and basal metabolic rate (BMR). Appendicular skeletal muscle mass index (ASMI) was estimated by bioelectrical impedance analysis (BIA) using the ASM/height^2^ equation. Low ASMI was defined according to the EWGSOP2 thresholds of ≤7.0 kg/m^2^ in men and ≤5.5 kg/m^2^ in women [24].

Measurements were performed in a standing position according to the manufacturer’s recommendations (i.e., bare feet, no metal objects, and rest before measurement). The InBody 270 analyzer has been reported to show acceptable accuracy and repeatability in adult populations, including older adults [25,26].

#### 2.3.2. Assessment of Nutritional Status

Nutritional status was assessed using the Mini Nutritional Assessment—Long Form (MNA-LF). The MNA total score was calculated, and participants were classified into the following malnutrition categories: <17 points; risk of malnutrition, 17–23.5 points; and normal nutritional status, ≥24 points [27]. The present study focused on malnutrition risk, with frailty and sarcopenia analyzed as distinct but overlapping geriatric constructs.

#### 2.3.3. Assessment of Depressive Symptoms

Depressive symptoms were assessed using the 30-item Geriatric Depression Scale (GDS-30) [28,29]. Participants were classified as having no significant depressive symptoms (0–9 points), mild to moderate depressive symptoms (10–19 points), or severe depressive symptoms (20–30 points). The total GDS score and GDS risk category were included in the analysis [30,31].

#### 2.3.4. Assessment of Physical and Functional Fitness

Physical performance was assessed using the Timed Up and Go (TUG) test. Participants were asked to stand up from a chair, walk 3 m, turn around, return to the chair, and sit down again. The time required to complete the task was recorded in seconds. A TUG time of ≥20 s was interpreted as indicating substantial mobility limitation and an increased risk of disability and frailty, in accordance with clinical recommendations [32].

Muscle strength was assessed by measuring handgrip strength (HGS) with a JAMAR^®^ PLUS+ handheld dynamometer (Performance Health Sammons Preston, Warrenville, IL, USA), in accordance with clinical practice guidelines for older adults [33]. Measurements were performed in a seated position and expressed in kilograms. Two measurements were taken for each upper limb, and the mean value was used for further analysis. Arm circumference was measured at the point of greatest circumference using a measuring tape and expressed in centimeters. According to the European Working Group on Sarcopenia in Older People (EWGSOP2) criteria, reduced handgrip strength was defined as <27 kg in men and <16 kg in women [34].

#### 2.3.5. Assessment of the Risk of Sarcopenia

In accordance with the guidelines of EWGSOP2, the SARC-F questionnaire was used to screen for the risk of sarcopenia. The questionnaire assesses five domains: strength, assistance with walking, rising from a chair, climbing stairs, and falls. A score of ≥4 was considered indicative of an increased risk of sarcopenia, whereas a score <4 indicated no significant risk [34].

#### 2.3.6. Assessment of the Risk of Frailty Syndrome

Frailty results from age-related declines in physiological reserve and multisystem function [35]. It was assessed using the Fried criteria, as described previously. Participants were classified as frail if at least three of the following five components were present: unintentional weight loss of at least 5 kg per year, exhaustion, weakness, slow gait speed, and low physical activity [36].

#### 2.3.7. Assessment of Appetite

The assessment of appetite was conducted using the Simplified Nutritional Appetite Questionnaire (SNAQ) and the Council on Nutrition Appetite Questionnaire (CNAQ). For the SNAQ, a cut-off score of ≤14 points was used, indicating a significant risk of losing ≥5% of body weight within 6 months (SNAQ risk). The CNAQ score was calculated as the sum of responses to its eight items, resulting in a total range of 8 to 40; scores below 28 were interpreted as indicating reduced appetite and an elevated risk of unintentional weight loss. In contrast, scores ranging from 28 to 40 points are considered within the normal range [37,38,39].

#### 2.3.8. Laboratory Parameters

The study procedure included the collection of venous blood samples for the assessment of biochemical and hematological parameters. Blood samples were collected in the morning, between 8:00 and 9:00 a.m., by qualified medical personnel after an overnight fast. Venous blood was drawn from the antecubital vein into tubes containing either a coagulation activator for serum preparation or dipotassium ethylenediaminetetraacetic acid (K2EDTA) for plasma and erythrocyte preparation.

A total of 30 laboratory parameters were evaluated in each participant.

Protein nutritional status was assessed based on transferrin (mg/dL), prealbumin (mg/dL), and albumin (g/dL). The lipid profile included HDL cholesterol, LDL cholesterol, non-HDL cholesterol, triglycerides, and total cholesterol, all expressed in mg/dL. Inflammatory status was assessed using C-reactive protein (CRP; mg/L).

A complete blood count was also performed and included the following parameters: white blood cells (WBC; K/µL), red blood cells (RBC; M/µL), hemoglobin (HGB; g/dL), hematocrit (HCT; %), mean corpuscular volume (MCV; fL), mean corpuscular hemoglobin (MCH; pg), mean corpuscular hemoglobin concentration (MCHC; g/dL), red cell distribution width (RDW; %), platelets (PLT; K/µL), plateletcrit (PCT; %), platelet distribution width (PDW; fL), and mean platelet volume (MPV; fL).

A blood smear was performed to determine the absolute and relative counts of neutrophils, lymphocytes, monocytes, eosinophils, and basophils. Absolute values were expressed as ×10^3^/µL, whereas relative values were expressed as percentages.

### 2.4. Statistical Analysis

All analyses were conducted at a significance level of 0.05. Categorical variables are reported as proportions and continuous variables as mean ± standard deviation. The analytic matrix comprised 105 individuals and 84 active variables grouped into nine domains: nutritional screening, sociodemographic characteristics, lifestyle, comorbidities, medication use, health assessment, risk assessment, body composition, and blood biomarkers. Fifty variables were quantitative and 34 were binary.

An unsupervised analytical framework combining Multiple Factor Analysis and Hierarchical Clustering on Principal Components was used to integrate quantitative and binary variables and identify homogeneous subgroups of older adults. Quantitative variables were centered and scaled within each domain. Binary variables were entered through complete disjunctive coding and handled within the categorical component of the Multiple Factor Analysis framework. In Multiple Factor Analysis, each domain was weighted by the inverse of the first eigenvalue of its separate analysis to balance contributions across domains and limit dominance by groups with more variables or stronger internal correlation. Five dimensions, accounting for 38.11% of total inertia, were retained for clustering.

**Figure A1:**
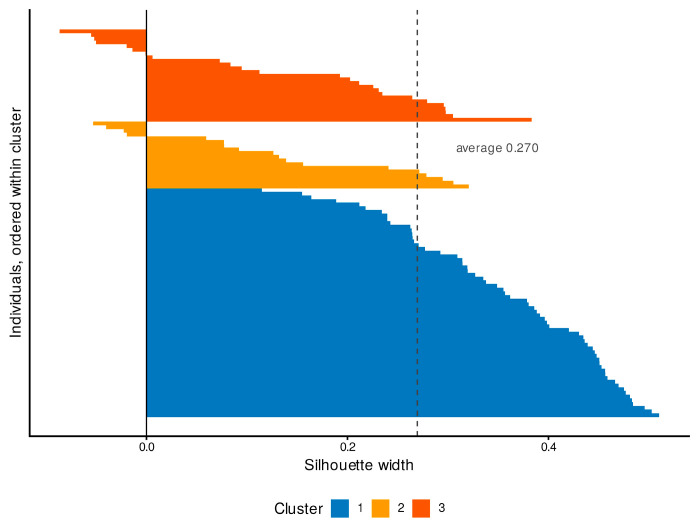
Silhouette analysis of the retained clustering solution.

Hierarchical clustering on principal components was then performed using Euclidean distances and Ward’s criterion, followed by k-means consolidation initialized at the hierarchical centroids. Three clusters were fixed a priori on the grounds of parsimony and clinical interpretability rather than selected as a data-driven optimum. Evidence bearing on this decision, including alternative cluster diagnostics, is reported in Section 3 and Appendix A (Table A1, Figure A1).

The Mini Nutritional Assessment score and MNA-defined malnutrition risk were included in the active set in the primary analysis. In a sensitivity analysis, both MNA variables were treated as supplementary and excluded from the construction of the clustering solution. This specification was adopted to assess the extent to which the clustering structure depended on direct inclusion of nutritional screening variables.

**Figure A2:**
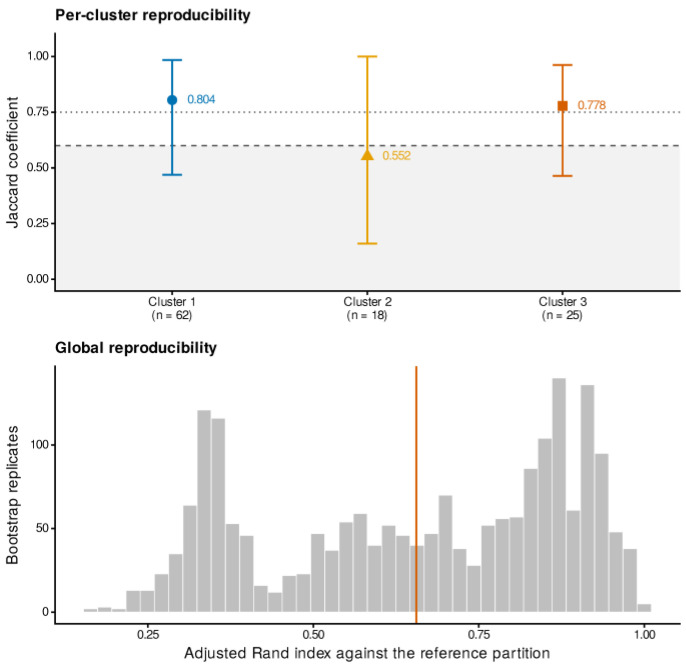
Bootstrap-based cluster stability assessed by adjusted Rand index and Jaccard coefficients.

**Figure A3:**
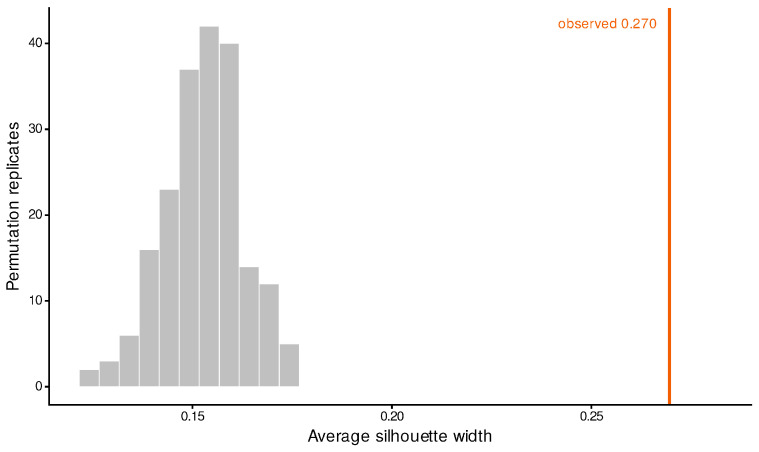
Observed cluster validation metrics versus permutation null distributions.

The analytic matrix was complete, and no imputation was required. Quantitative variables were examined for distributional shape, but no transformations were applied because the analysis relied on the correlation structure of standardized variables. Binary variables were entered through complete disjunctive coding, with one indicator column per level. This weighting is the mechanism by which variable redundancy is handled, and it is the reason no variable selection procedure was applied. Several body-composition measures are strongly collinear, deriving from a single bioimpedance model; a data-driven selection would have added a further layer of post-selection inference to the analysis. Data auditing included a systematic outlier review and correction of three recording errors in the hematology panel. Cluster quality was assessed using silhouette widths, bootstrap stability over 2000 resamples, the adjusted Rand index, per-cluster Jaccard coefficients, and the between-cluster share of inertia. To calibrate these quantities against a procedural null, the full Multiple Factor Analysis–Hierarchical Clustering on Principal Components pipeline was also applied to 200 permuted datasets in which marginal distributions were preserved but multivariable association was destroyed. Full validation results and permutation-based calibration are presented in Appendix A (Figure A2 and Figure A3).

**Figure A4:**
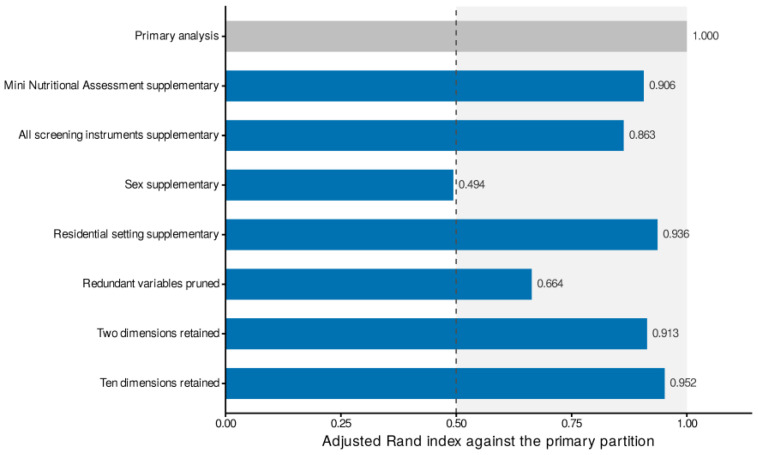
Three further families of sensitivity analysis.

Sensitivity analyses were conducted to assess the robustness of the clustering solution. These included repeating the analysis with the Mini Nutritional Assessment score and risk category treated as supplementary variables, excluding all four screening instruments from the active set, treating sex and residential setting as supplementary variables, refitting the pipeline within strata defined by sex and setting, pruning strongly coupled variables, and varying the number of retained dimensions from two to ten. Agreement with the primary partition and the persistence of the association with malnutrition risk across these specifications are summarized in Appendix A (Table A2 and Table A3, Figure A4).

**Figure A5:**
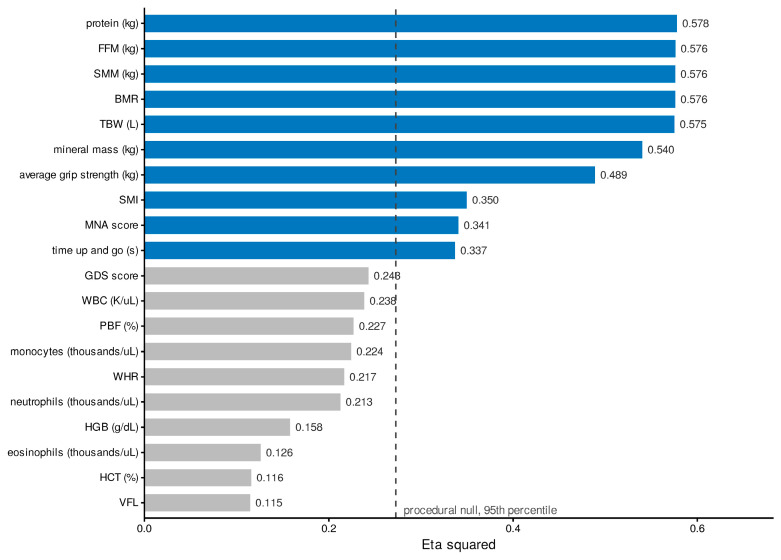
Eta-squared effect sizes for continuous variables with the procedural null threshold.

Because the clusters were constructed from the same variables later used for characterization, post-clustering comparisons were treated as descriptive rather than confirmatory. Effect sizes were reported as eta squared for continuous variables and Cramer’s V for categorical variables, and *p* values were retained as an ordering device only. Multiplicity was addressed using the Benjamini–Hochberg false discovery rate procedure across the full family of 84 comparisons, with Bonferroni-adjusted results reported as a stricter reference. Empirical calibration of apparent effect sizes using 200 permuted datasets is presented in Appendix A (Table A4, Figure A5).

A composite nutritional risk index was defined as the count of positive screens across the Mini Nutritional Assessment, the Simplified Nutritional Appetite Questionnaire, the Council on Nutrition Appetite Questionnaire, and the SARC-F, yielding scores from 0 to 4. Internal consistency and inter-item relationships of this index are reported in Appendix A (Table A4).

Internal predictive validation of malnutrition risk discrimination was assessed using repeated stratified five-fold cross-validation. Predictor sets were compared both in the primary framework and in analyses treating Mini Nutritional Assessment variables as supplementary to further reduce circularity. The full predictive validation protocol and results are presented in Appendix A (Table A4, Figure A5).

Analyses were conducted using the R Statistical language (version 4.3.3; R Core Team, 2024) on Windows 11 Pro 64 bit (build 22631), using the following packages: rio (version 1.2.1), factoextra (version 1.0.7), FactoMineR (version 2.11), sjPlot (version 2.8.15), report (version 0.5.8), gtsummary (version 1.7.2), ggplot2 (version 3.5.0), and dplyr (version 1.1.4).

Further methodological details, including validation, sensitivity analyses, data preprocessing, multiplicity handling, and internal predictive assessment, are provided in Appendix A.1.

## 3. Results

### 3.1. Hierarchical Clustering on Principal Components

**Figure 2:**
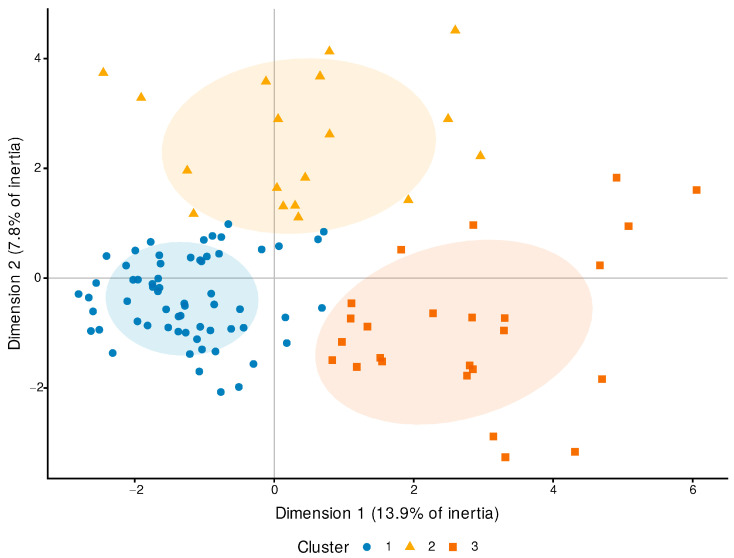
Scatter plot of hierarchical clustering on principal components (HCPC) for three clusters, projected onto the first two principal components (dimension 1: 13.9% variance, dimension 2: 7.8% variance).

The HCPC analysis of the 105 patients and 84 parameters (Table 1) yielded a three-cluster solution, shown in a two-dimensional scatter plot based on the first two principal components, which explained 13.9% and 7.8% of the total variance, respectively (Figure 2). The clusters comprised *n*1 = 62, *n*2 = 18, and *n*3 = 25 individuals. The analytic matrix was complete, with no missing values among the 8820 cells. In Figure 2, concentration ellipses are shown instead of convex hulls, which are more influenced by extreme observations and may exaggerate apparent separation. The overlap between clusters therefore offers a more faithful representation of the data. The validation results in Section 3.2 indicate that the structure is present but only weakly separated, rather than reflecting robust stratification into homogeneous subgroups.

### 3.2. Cluster Validation

The retained solution shows weak structure (average silhouette width 0.270; Figure A1, Appendix A) and moderate stability (adjusted Rand index 0.656; Figure A2, Appendix A). Cluster 2 is least stable (Jaccard 0.552) and is therefore qualified throughout. The between-cluster share of inertia is 38.5%.

The observed validation metrics exceeded the entire permutation-based reference distribution (permutation *p* = 0.005 for all; Figure A3 in Appendix A), indicating a real but weakly separated structure. Full validation quantities are reported in Table 2.

Released from the original constraint, the automatic criterion preferred five clusters, whereas silhouette width and bootstrap agreement were highest for two clusters. Three clusters were retained a priori for parsimony and clinical interpretability, and the supporting and opposing evidence is reported in Table 2.

### 3.3. Association of Categorical and Quantitative Variables with Clusters

Chi-squared tests showed that 23 of 34 categorical variables were significantly associated with cluster membership (*p* < 0.05), with the strongest associations observed for sex, frailty risk, residential care, malnutrition risk and polypharmacy. The detailed data are presented in Table 3.

Among the quantitative variables, 27 of 50 were significantly related to cluster allocation (*p* < 0.05), with η^2^ values ranging from 0.06 to 0.58. The highest effect sizes were found for protein mass, fat-free mass, basal metabolic rate, skeletal muscle mass, total body water, and mineral mass (η^2^ > 0.54, *p* < 0.001), indicating strong discrimination between clusters. Moderate associations were observed for average grip strength, MNA score, ASMI, and the Timed Up and Go test (η^2^ = 0.33–0.48, *p* < 0.001), whereas BMI, lymphocyte count, non-HDL cholesterol, and red cell distribution width showed weaker but still significant associations (η^2^ < 0.08, *p* = 0.01–0.05). The cluster exhibiting a higher prevalence of malnutrition risk should be interpreted as a profile associated with nutritional vulnerability. The distribution of MNA score and MNA risk across clusters provides descriptive characterization of the identified profiles. Table 4 presents the detailed data.

### 3.4. Nutritional and Geriatric Characteristics of the Three Clusters

#### 3.4.1. Cluster 1—Relatively Preserved Nutritional and Geriatric Health Phenotype

Cluster 1 comprised 62 older adults and was associated with a lower malnutrition risk and consisted exclusively of women. It was also characterized by lower rates of frailty, residential care, depressive symptoms, polypharmacy, major comorbidities, smoking, and recent hospitalization, together with better functional status and higher educational attainment. Comprehensive details are provided in Appendix B, Table A5.

Cluster 1 showed higher MNA scores than the overall sample and a generally more favorable profile, with lower CRP, lower WHR, lower GDS scores, and faster TUG times. It also showed higher LDL, total, non-HDL and HDL cholesterol levels. Full details are provided in Appendix B, Table A6.

#### 3.4.2. Cluster 2—Predominantly Male Phenotype with Preserved Muscle Mass and Functional Reserve

Cluster 2 comprised 18 older adults, including 17 men, and showed no significant difference in malnutrition risk or mean MNA score compared with the overall sample. The cluster was predominantly male and was characterized by lower use of painkillers and anti-inflammatory medications, as well as lower alcohol consumption, than in the global sample. Detailed information is provided in Appendix B, Table A7. The role of sex is explained in Section 3.5. This finding warrants a cautious interpretation of the cluster as a possible independent clinical phenotype.

Cluster 2 showed higher protein mass, skeletal muscle mass, fat-free mass, basal metabolic rate, total body water, mineral mass, grip strength, and appendicular skeletal muscle index than the overall sample. It also had a higher waist-to-hip ratio, monocyte and neutrophil counts, hemoglobin, and hematocrit, but lower visceral fat, HDL cholesterol, and body fat percentage. Detailed information is provided in Appendix B, Table A8.

#### 3.4.3. Cluster 3—Nutritionally Vulnerable and Frail Phenotype with Multimorbidity and Functional Impairment

Cluster 3 was associated with a significantly higher risk of malnutrition according to the MNA and was characterized by frailty, residential care, polypharmacy, multimorbidity, functional impairment, and greater psychological distress. Appendix B, Table A9 provides detailed information.

Cluster 3 showed slower Timed Up and Go performance, higher GDS scores, greater adiposity, higher BMI, lower grip strength, and elevated counts of several hematological markers, alongside lower lipid, hematocrit, and hemoglobin values. Full details are provided in Table A10 of Appendix B.

### 3.5. Sensitivity Analyses

Refitting the pipeline with the Mini Nutritional Assessment treated as supplementary yielded a partition nearly identical to the primary solution, and the association with malnutrition risk remained essentially unchanged. Excluding all four screening instruments simultaneously led to the same conclusion, suggesting that the result is not driven by circularity. Full results are reported in Table 5.

Sex was the main organizing factor, and treating it as supplementary changed the partition, although the association with malnutrition risk persisted. Subgroup refitting gave adjusted Rand indices of 0.826 for community-dwelling participants, 0.430 for residential care, 0.361 for women, and 0.420 for men, although only the community-dwelling result is interpretable. Pruning strongly coupled variables slightly changed the partition, but the association with malnutrition risk remained unchanged. Full sensitivity analyses are reported in Table A2 and Figure A4 in Appendix A. All detailed results are provided in Appendix B.2.

The full multiple-testing and permutation-calibrated results are presented in Appendix A, with the ten variables exceeding the procedural-null threshold highlighted in Table A3 and Figure A5. The composite index is therefore reported as a count of positive screens rather than as a latent severity measure, with full psychometric characteristics presented in Table A2 (Appendix A) and further described in Appendix B.2. Internal validation suggested an association between cluster membership and malnutrition risk, although this finding should be interpreted cautiously and does not by itself demonstrate clinical utility (Table A4, Appendix A).

## 4. Discussion

In this study, HCPC was applied to multidimensional health data from older adults to examine malnutrition risk within a broader geriatric context. Three clinically interpretable clusters were identified, with increased malnutrition risk confined to one cluster characterized by greater functional decline, frailty, chronic disease burden, and other adverse health-related features. Accordingly, the clusters should be viewed as descriptive profiles of heterogeneity rather than definitive or causal categories [21,40].

Although comprehensive geriatric assessment is well established in the care of older adults [41,42,43,44], its integration with data-driven clustering remains relatively limited [45,46]. Our findings underscore the value of employing an unsupervised multivariate method on a clinically rich geriatric dataset to obtain a more nuanced profile of nutritional vulnerability in older adults. This highlights the potential of phenotype-based stratification for exploring the multidimensional and overlapping nature of frailty, multimorbidity, and nutritional vulnerability [47]. As a future direction, scalable, non-invasive digital tools may complement multidimensional phenotyping, particularly given the modifiable role of oral health in malnutrition pathways [48]. The cluster structure observed in this study suggests that geriatric vulnerability may increase along a continuum rather than form clearly separate groups, which is consistent with the concept of frailty as a dynamic spectrum of vulnerability and resilience [49].

Cluster 1 appears to represent a relatively preserved phenotype, with lower geriatric vulnerability and greater physiological reserve. Higher total cholesterol and lower inflammatory status may be consistent with a comparatively more favorable clinical profile [50,51,52]. Cluster 1 was predominantly female and showed the best nutritional status, despite evidence that women are generally at higher risk of malnutrition in older populations [53,54,55,56,57]. In our study, the cluster may reflect a more favorable phenotype, with better physical function, lower inflammatory burden, and lower disease burden, which may be associated with the lower observed risk in this subgroup. This interpretation should remain cautious, however, as older adults may still be vulnerable to malnutrition and functional decline even in the absence of overt deficits, and periodic monitoring remains warranted [1,4,58,59].

Cluster 2, which was composed mainly of men, may indicate a potentially intermediate clinical phenotype, with relatively preserved features alongside some markers of vulnerability. This interpretation is consistent with evidence linking malnutrition risk in older adults to multimorbidity, polypharmacy, physical limitations, and broader geriatric vulnerability, which tend to develop gradually rather than abruptly [60,61,62,63,64,65].

Cluster 3 represents the subgroup with the highest malnutrition risk and the greatest overall health vulnerability. It was characterized by universal malnutrition risk according to the MNA, impaired physical function, higher frailty risk, depression and residential care. Participants exhibit substantial multimorbidity, including diabetes mellitus, hypertension, neurological conditions, and heart disease, alongside polypharmacy, high hospitalization rates and a relatively high prevalence of hyperthyroidism, which should be interpreted cautiously as a descriptive finding. Compared with the overall sample, this subgroup showed higher BMI, greater visceral and total fat, elevated inflammatory markers, and lower cholesterol and hemoglobin levels.

This is consistent with prior studies showing that unsupervised methods can identify clinically relevant nutritional phenotypes in older adults, including individuals at risk of malnutrition who may not be fully captured by conventional screening tools [66,67,68]. Our preliminary results extend this work by integrating functional, biological, and clinical variables to better identify individuals at risk of malnutrition or poor nutritional status.

These findings highlight the need for a multidimensional approach to assessing malnutrition risk in older adults, particularly as they align with broader literature showing that such risk is strongly associated with poor physical function, disease burden, and recent hospitalizations [69,70], while higher grip strength and muscle mass serve as protective markers against nutritional risk and frailty [71,72,73,74,75,76]. The contrast between Cluster 3 and Cluster 2 shows that retained functional reserve and lower disease burden may be critical determinants of nutritional status. This aligns with prior studies reporting associations between nutritional risk and multiple physical and clinical domains [77,78]. These observations suggest that maintaining high muscle mass and physical strength may protect against malnutrition in the elderly [79,80]. However, these findings should be interpreted cautiously due to gender imbalances.

The observed association between malnutrition risk and frailty in this study is consistent with the findings reported in the previous literature [72,81]. Frailty and malnutrition have been shown to share common biological and clinical pathways, including sarcopenia, inflammation, low energy intake, and reduced activity [82]. In practice, the presence of frailty can increase the risk of malnutrition, which can in turn exacerbate the condition. This is due to a combination of factors, including impaired mobility, reduced appetite, and decreased ability to prepare meals [83]. Conversely, malnutrition can lead to accelerated frailty by worsening muscle loss and weakness [84]. The present findings appear to support the view that these conditions should be assessed together in geriatric care [85,86].

Physical function was one of the domains that differentiated the identified phenotypes. The more vulnerable clusters showed poorer Timed Up and Go performance and lower handgrip strength, together with less favorable muscle-related body composition measures. This pattern is consistent with previous studies indicating that reduced mobility and muscle weakness are important indicators of nutritional decline in older adults [79,87,88,89]. Handgrip strength and gait-related measures may be particularly informative, as they reflect not only muscle mass but also neuromuscular performance and overall functional reserve [90,91,92]. In the present study, these functional impairments co-occurred with other markers of vulnerability within distinct clinical profiles, supporting the relevance of multidimensional assessment in the evaluation of nutritional risk in older adults [76,93].

The disparities in body composition ascertained in this study are consistent with the clinical interpretations of the identified groups [94]. The subjects exhibiting a more vulnerable phenotype demonstrated less favorable patterns of fat distribution, visceral adiposity, lean tissue, and muscle-related indices, suggesting a compromised nutritional and functional profile. These findings are clinically relevant because, in older adults, excess adiposity does not necessarily indicate adequate nutritional status [95]. Rather, sarcopenic obesity may coexist with insufficient protein intake [96,97] and progressive muscle loss [98,99]. Conversely, a significant decrease in lean mass and fat-free mass may indicate a more advanced nutritional compromise [100]. The present study may extend the available literature by demonstrating that such abnormalities cluster within distinct phenotypes [101]. This finding suggests that body composition provides meaningful information beyond BMI alone, which may overlook hidden nutritional risk [102]. This study emphasizes integrating body composition evaluation into research to enhance early malnutrition detection in older adults.

Laboratory variables also contributed to the descriptive characterization of the identified phenotypes [21,103,104]. The observed differences in hemoglobin-related parameters, lipid fractions, inflammatory markers, and other hematological indices may reflect broader vulnerability patterns in older adults rather than malnutrition risk alone [105,106]. This is consistent with Zang et al.’s meta-analysis, which found lower levels of albumin, hemoglobin, total cholesterol, prealbumin, and total protein in individuals at high risk of malnutrition classified by the MNA [107]. Similarly, previous studies have shown that poorer nutritional status in older adults is associated with lower hemoglobin levels [108,109], lower total cholesterol concentrations [110] and greater inflammatory burden [111]. Laboratory markers may contribute to the descriptive characterization of malnutrition-related profiles [104]. However, in the absence of reference-range analysis, these findings should be interpreted with caution and regarded as descriptive rather than clinically diagnostic indicators of malnutrition risk [112]. The co-occurrence of hematological, inflammatory, and lipid-related differences across clusters may reflect broader vulnerability profiles in older adults [113,114]. Overall, the observed laboratory differences may be considered part of the descriptive vulnerability profiles identified in this study rather than independent diagnostic markers of malnutrition risk.

In the present study, medication burden clearly differentiated more vulnerable subgroups, with the third cluster showing a higher prevalence of polypharmacy and more frequent use of antihypertensive, diuretic, anticoagulant, and antidepressant therapies. These observations are consistent with the extant literature indicating that polypharmacy is closely linked to malnutrition risk [115,116,117] and broader geriatric syndromes, including frailty [118] and multimorbidity [119]. Notably, the findings suggest that medication burden may serve not only as a measure of disease complexity but also as an informative marker of distinct vulnerability phenotypes in older adults. These findings highlight the potential value of incorporating systematic medication reviews into nutritional assessments, particularly among older adults presenting with frailty.

Psychological vulnerability was most evident in phenotypic groups at elevated risk of malnutrition, particularly in clusters 2 and 3, where residential care and depression were more prevalent than in cluster 1. This pattern suggests that malnutrition vulnerability in older adults may reflect a broader psychosocial burden rather than impaired nutritional status alone [120,121,122]. This interpretation is particularly relevant to cluster 3, which showed a higher risk of malnutrition together with a greater prevalence of residential care and depressive symptoms. Consistent with prior evidence that both depression and residential care are independently associated with frailty, and that their co-occurrence further increases frailty risk, this cluster may reflect a broader frailty-related phenotype rather than nutritional vulnerability alone [123]. This phenomenon has significant implications for self-management and functional independence in older adults [124].

Our findings suggest that unsupervised clustering may be useful for describing multidimensional patterns of malnutrition vulnerability in older adults. Given the exploratory and cross-sectional design, the results should be interpreted cautiously as preliminary findings. Nonetheless, the findings highlight clinically relevant heterogeneity across phenotypic profiles and may provide a basis for future research on nutritional assessment and intervention.

### Limitations and Strengths of the Study

Several limitations of this study should be acknowledged. The cross-sectional design precludes causal inference and the modest sample size may limit generalizability. The clusters should be interpreted cautiously because MNA was included among the active variables and the overall structure was only weakly separated. The observed sex-related differences in body composition and biochemistry may partly shape the identified phenotypes, although the sample is too limited to support sex-stratified phenotyping. The clustering solution was moderately sensitive to the coupled body-composition block, but the main association with malnutrition risk remained stable. Although sensitivity analyses suggested that the association between Cluster 3 and malnutrition risk was robust when MNA was treated as supplementary and when screening instruments were excluded, the profiles remain descriptive rather than definitive. The data did not include factors such as dietary intake, socio-economic status, social support, or oral health, all of which may influence malnutrition risk and warrant further investigation in future research. Due to the preliminary nature of the study, the sample was not intended to be representative of the general older adult population. Recruitment was less balanced across key subgroups, particularly men, participants with different living arrangements, and adults over 70 years of age, which may have introduced some selection bias. Instead, it was designed to provide an initial evaluation of a multidimensional, data-driven approach to malnutrition risk phenotyping, in a restricted group of ambulatory, clinically stable older adults. Accordingly, this clustering should be considered exploratory, and the findings should be interpreted with caution.

Despite its limitations, this study has notable strengths. The findings may contribute to a more nuanced understanding of malnutrition risk in older adults by highlighting multidimensional vulnerability profiles. The use of unsupervised machine learning, including MFA and HCPC, helped identify distinct clinical and nutritional patterns within the older adult population. Compared with traditional one-dimensional analyses, this multidimensional approach may provide a more comprehensive characterization of nutritional vulnerability in older adults.

Further research is needed to validate the stability of these clusters and their predictive clinical value in larger, more representative populations using prospective study designs. This should include a prospective cohort follow-up study to assess whether clustering-based phenotypic classification predicts clinically meaningful outcomes such as falls, hospitalization, and functional decline in older adults. Future studies should also examine whether phenotype-based stratification improves malnutrition risk prediction and supports more individualized management in older adults.

## 5. Conclusions

In summary, hierarchical clustering revealed three clinically distinct phenotypes among older adults, with one cluster showing increased malnutrition risk and broader multidimensional vulnerability, including frailty, functional decline, medication burden, residential care, and unfavorable body composition.

This exploratory study suggests that unsupervised machine learning with a multidimensional approach may help characterize nutritional profiles associated with malnutrition risk in older adults. Sensitivity analyses supported the robustness of the clustering structure, although sex influenced it to some extent and one small cluster was less stable. These findings should therefore be regarded as exploratory rather than confirmatory. Prospective validation in independent cohorts is needed before clinical utility can be established.

## Figures and Tables

**Table 1:** Overview of characteristics in older adults (*N* = 105): Proportions for categorical data and means ± SD for continuous data.

Characteristic	Number of Events, % or Mean ± SD	Unit
Demographics		
Female sex	83, 79.05	%
Age < 70 years	60, 57.14	%
Residential care	24, 22.90	%
Higher education	49, 46.67	%
Complete dentition	26, 24.76	%
Health conditions		
Serious illnesses (past year)	13, 12.38	%
Hospitalization (past year)	17, 16.19	%
Current smoking	15, 14.29	%
Alcohol consumption	66, 62.86	%
Regular meal consumption	83, 79.05	%
Hypertension	68, 64.76	%
Diabetes mellitus	15, 14.29	%
Gastroesophageal reflux disease (GERD)	25, 23.81	%
Heart disease	28, 26.67	%
Hypothyroidism	13, 12.38	%
Hyperthyroidism	23, 21.90	%
Degenerative spine/joint disease	22, 20.95	%
Medication use		
Polypharmacy	37, 35.24	%
Painkiller use	62, 59.05	%
Statin use	35, 33.33	%
Antidiabetic medication use	18, 17.14	%
Neurological conditions	23, 21.90	%
Anti-inflammatory medication use	40, 38.10	%
Anticoagulant use	23, 21.90	%
Antihypertensive medication use	65, 61.90	%
Antidepressant use	15, 14.29	%
Hypothyroidism medication use	12, 11.43	%
Diuretic use	24, 22.86	%
Nutritional and functional risk assessments		
Frailty Risk	37, 35.24	%
Geriatric Depression Scale (GDS) risk	37, 35.24	%
Council on Nutrition Appetite Questionnaire (CNAQ) risk	57, 54.29	%
Simplified Nutritional Appetite Questionnaire (SNAQ) risk	36, 34.29	%
SARC-F risk (sarcopenia)	30, 28.57	%
Waist-to-hip ratio	0.91 ± 0.07	Unitless
Geriatric Depression Scale (GDS)	7.20 ± 5.34	Score
Timed Up and Go test (s)	12.05 ± 7.77	seconds
Average grip strength	19.87 ± 7.72	kg
Council on Nutrition Appetite Questionnaire (CNAQ)	14.05 ± 1.89	Score
Simplified Nutritional Appetite Questionnaire (SNAQ)	15.94 ± 2.28	Score
SARC-F (Sarcopenia)	2.31 ± 2.47	Score
Anthropometric measures		
Total body water	33.39 ± 6.09	L
Protein mass	8.88 ± 1.63	kg
Mineral mass	3.17 ± 0.54	kg
Body fat mass	28.32 ± 10.27	kg
Fat-free mass	45.44 ± 8.24	kg
Skeletal muscle mass	24.79 ± 4.93	kg
Appendicular skeletal muscle index	9.51 ± 1.22	kg/m^2^
Body mass index (BMI)	28.58 ± 5.44	kg/m^2^
Percent body fat	37.70 ± 8.24	%
Basal metabolic rate	1351.50 ± 177.79	kcal/day
Visceral fat level	13.80 ± 5.24	Unitless
Laboratory parameters		
Transferrin	231.65 ± 32.44	mg/dL
Prealbumin	26.06 ± 4.78	mg/dL
HDL cholesterol	68.60 ± 18.08	mg/dL
LDL cholesterol	119.21 ± 39.67	mg/dL
Non-HDL cholesterol	142.31 ± 42.56	mg/dL
Triglycerides	116.08 ± 54.35	mg/dL
Total cholesterol	210.89 ± 48.11	mg/dL
Albumin	4.72 ± 0.36	g/dL
C-Reactive protein (CRP)	4.03 ± 8.93	mg/L
White blood cells (WBC)	6.85 ± 7.36	K/µL
Red blood cells (RBC)	4.53 ± 0.47	M/µL
Hemoglobin (HGB)	13.64 ± 1.17	g/dL
Hematocrit (HCT)	41.84 ± 3.50	%
Mean corpuscular volume (MCV)	92.79 ± 6.22	fL
Mean corpuscular hemoglobin (MCH)	30.24 ± 2.17	pg
Mean corpuscular hemoglobin concentration (MCHC)	32.60 ± 0.80	g/dL
Red cell distribution width (RDW)	13.76 ± 1.13	%
Platelets (PLT)	236.36 ± 63.20	K/µL
Plateletcrit (PCT)	0.23 ± 0.06	%
Platelet distribution width (PDW)	15.03 ± 2.50	fL
Mean platelet volume (MPV)	10.42 ± 8.04	fL
Neutrophils	3.52 ± 1.28	×10^3^/µL
Lymphocytes	2.00 ± 1.25	×10^3^/µL
Monocytes	0.42 ± 0.19	×10^3^/µL
Eosinophils	0.18 ± 0.11	×10^3^/µL
Basophils	0.05 ± 0.02	×10^3^/µL
Neutrophils (%)	57.12 ± 9.67	%
Lymphocytes (%)	32.41 ± 8.75	%
Monocytes (%)	6.84 ± 2.16	%
Eosinophils (%)	2.81 ± 1.32	%
Basophils (%)	0.81 ± 0.37	%
MNA assessment (primary outcome)		
Malnutrition risk according to MNA	50, 47.62	*N*, %
MNA (score)	23.82 ± 3.22	Score

**Table 2:** Evidence bearing on the number of clusters and validation of the retained solution.

Quantity	Value	Threshold or Reference
Average silhouette width, k = 2	0.313	—
Average silhouette width, k = 3 (retained)	0.270	<0.25 weak; >0.50 moderate
Average silhouette width, k = 4	0.255	—
Average silhouette width, k = 5	0.229	—
Preference of the automatic criterion, unconstrained	k = 5	—
Bootstrap adjusted Rand index, k = 2	0.841	—
Bootstrap adjusted Rand index, k = 3 (retained)	0.656 (0.274–0.968)	>0.50 substantial
Silhouette width, cluster 1	0.361	—
Silhouette width, cluster 2	0.135	—
Silhouette width, cluster 3	0.140	—
Individuals with negative silhouette width	11 of 105	—
Bootstrap Jaccard, cluster 1 (*n* = 62)	0.804	<0.60 untrustworthy; >0.75 stable
Bootstrap Jaccard, cluster 2 (*n* = 18)	0.552	<0.60 untrustworthy; >0.75 stable
Bootstrap Jaccard, cluster 3 (*n* = 25)	0.778	<0.60 untrustworthy; >0.75 stable
Cluster 2 dissolution rate across resamples	47.6%	—
Between-cluster share of inertia	38.5%	null 26.5% (24.5–28.5)
Average silhouette width against the permutation null	0.270	null 0.153 (0.133–0.172); *p* = 0.005
Inertia of dimension 1 against the permutation null	13.90%	null 4.22% (3.95–4.57); *p* = 0.005

Note: Bootstrap quantities derive from 2000 resamples; the permutation null derives from 200 refits of the complete pipeline on datasets in which every variable was permuted independently, preserving all marginal distributions while destroying association. Three clusters were retained a priori on grounds of parsimony and interpretability; the table reports the evidence favouring and opposing that choice without selection.

**Table 3:** Categorical variables by cluster, degrees of freedom (*df*) = 2.

Variable	*p*-Value
Female	<0.001
Risk of frailty	<0.001
Residential care	<0.001
Mini Nutritional Assessment (MNA) Risk	<0.001
Polypharmacy	<0.001
Anticoagulants	<0.001
Neurological conditions	<0.001
Diuretics intake	<0.001
Diabetes Mellitus (DM)	<0.001
Higher Education	<0.001
Antidiabetic medication intake	<0.001
Alcohol consumption	<0.001
Smoking	<0.001
Anti-inflammatory medication intake	<0.001
Geriatric Depression Scale (GDS) Risk	0.001
Hospitalization in the past year	0.002
Antidepressant’s intake	0.003
Hypertension	0.004
Heart disease	0.004
Age below 70 years	0.004
Antihypertensive medication intake	0.005
Hyperthyroidism	0.005
Painkiller’s intake	0.015

Note: *p*-values are Benjamini–Hochberg adjusted across the joint family of 84 comparisons and are reported as descriptive ordering quantities rather than as confirmatory tests, because the variables tabulated were also used to construct the clusters. Cramer’s V is the effect size on which interpretation rests. Of 52 nominally significant comparisons, 50 survive false discovery rate control and 32 survive Bonferroni correction.

**Table 4:** Eta squared values and false discovery rate-adjusted *p*-values for quantitative variables by cluster.

Variable	η^2^	*p*-Value
Strongest associations		
Protein mass (kg)	0.58	<0.001
Fat-free mass (FFM, kg)	0.58	<0.001
Basal metabolic rate (BMR)	0.58	<0.001
Skeletal muscle mass (SMM, kg)	0.58	<0.001
Total body water (TBW, L)	0.58	<0.001
Mineral mass (kg)	0.54	<0.001
Strong and moderate associations		
Average grip strength (kg)	0.48	<0.001
Mini Nutritional Assessment (MNA) score	0.36	<0.001
Appendicular skeletal muscle index (ASMI)	0.35	<0.001
Timed Up and Go test (s)	0.33	<0.001
Percent body fat (PBF, %)	0.23	<0.001
Geriatric Depression Scale (GDS) score	0.23	<0.001
Waist-to-hip ratio (WHR)	0.21	<0.001
Neutrophils (×10^3^/μL)	0.21	<0.001
Monocytes (×10^3^/μL)	0.20	<0.001
Hemoglobin (HGB, g/dL)	0.15	<0.001
Visceral fat level (VFL)	0.13	<0.001
Eosinophils (×10^3^/μL)	0.12	0.001
Hematocrit (HCT, %)	0.11	0.003
Basophils (%)	0.11	0.003
Low-density lipoprotein cholesterol (LDL, mg/dL)	0.10	0.004
High-density lipoprotein cholesterol (HDL, mg/dL)	0.10	0.006
Total cholesterol (mg/dL)	0.10	0.006
Weak associations		
Body fat mass (BFM, kg)	0.08	0.017
Non-HDL cholesterol (mg/dL)	0.07	0.023
Red cell distribution width (RDW, %)	0.07	0.027
Body mass index (BMI, kg/m^2^)	0.06	0.043
Lymphocytes (×10^3^/μL)	0.06	0.048

Note: These comparisons are descriptive, as the clusters were constructed by maximizing separation on the same variables; accordingly, η^2^, rather than the *p*-value, carries the main interpretive weight. Variables exceeding the 95th percentile of the procedural null distribution (η^2^ > 0.273; Table A3 and Figure A5, Appendix A) are the least susceptible to post-selection inflation.

**Table 5:** Sensitivity of the partition and of the association with malnutrition risk to the removal of the screening instruments from the active variable set.

Specification	Active Variables	Cluster Sizes	Agreement with the Primary Partition (ARI)	At Risk by Cluster (%)	Cramér’s V	*p*
Primary analysis (all instruments active)	84	62/18/25	1.000	27.4/44.4/100.0	0.599	6.5 × 10^−9^
Mini Nutritional Assessment supplementary	82	65/18/22	0.906	30.8/44.4/100.0	0.549	1.3 × 10^−7^
All four instruments supplementary	76	66/17/22	0.863	31.8/41.2/100.0	0.544	1.8 × 10^−7^

Note: Supplementary variables are projected into the factor space without contributing to its construction. The association with malnutrition risk retains 91.6 per cent of its magnitude when the Mini Nutritional Assessment is held out and 90.8 per cent when all four instruments are held out, establishing that it is not an artefact of the instrument’s inclusion among the active variables.

## Data Availability

The original data presented in the study are openly available in Polish Platform of Medical Research at https://doi.org/10.60945/ayh2-tk84.

## Abbreviations

MNA	Mini Nutritional Assessment
NRS 2002	Nutritional Risk Score
HCPC	Hierarchical clustering on principal components
MFA	Multiple Factor Analysis
CGA	Comprehensive Geriatric Assessment
ML	Machine Learning
PCA	Principal Component Analysis
BMI	Body mass index
WHR	Waist-to-hip ratio
MAC	Upper arm circumference
CC	Calf circumference
DM	Diabetes mellitus
GERD	Gastro-esophageal reflux disease
BIA	Bioelectrical impedance analysis
VFL	Visceral fat level
TBW	Total body water
FFM	Fat-free mass
BFM	Body fat mass
SMM	Skeletal muscle mass
ASMI	Appendicular skeletal muscle index
PBF	Percentage body fat
BMR	Basal metabolic rate
GDS	Geriatric depression scale
TUG	Timed Up and Go
HGS	Handgrip strength
EWGSOP2	European Working Group on Sarcopenia in Older People
K2EDTA	Dipotassium ethylenediaminetetraacetic acid
SARC-F	Strength, Assistance with Walking, Rising from a Chair, Climbing Stairs, and Falls (SARC-F)
SNAQ	Simplified Nutritional Appetite Questionnaire
CNAQ	Council on Nutrition Appetite Questionnaire
HGB	Hemoglobin
HCT	Hematocrit
RDW	Red cell distribution width
HDL	High density lipoprotein cholesterol
LDL	Low density lipoprotein cholesterol
CRP	C-reactive protein

## Appendix A

### Appendix A.1

**Table A1:** Specification of the Multiple Factor Analysis: group structure, weighting and contribution to the first two dimensions.

Group	Variables	Type	Inertia of the Group’s First Axis (%)	Divisor	Contribution to Dimension 1 (%)	Contribution to Dimension 2 (%)
Nutritional screening	2	Mixed	—	1.155	8.72	4.15
Sociodemographic	5	Categorical	—	1.414	13.05	9.88
Lifestyle	5	Categorical	—	1.414	9.51	12.40
Comorbidities	7	Categorical	—	1.414	11.86	14.62
Medication	11	Categorical	—	1.414	16.03	13.28
Health assessment	7	Quantitative	32.26	1.503	16.44	11.30
Risk assessment	5	Categorical	—	1.414	14.98	12.06
Body composition	11	Quantitative	63.30	2.639	0.86	9.42
Blood biomarkers	31	Quantitative	15.72	2.208	8.55	12.89

Note: The divisor is the square root of the first eigenvalue of each group’s separate analysis. Dividing by it equalizes the maximum axial inertia of every group before the global analysis, so that a group cannot dominate through the number or the intercorrelation of its variables. Body composition illustrates the mechanism: it is the most internally redundant group and receives the heaviest divisor, with the consequence that its 11 variables contribute 0.86 per cent to the first dimension. Inertia of the first axis is reported for quaSntitative groups only, for which it is directly interpretable.

**Table A2:** Sensitivity of the partition across specifications of the variable set, the sample composition and the retained dimensionality.

Specification	Detail	Agreement with the Primary Partition (ARI)	At Risk by Cluster (%)	Cramér’s V
Primary analysis	84 active variables, 5 dimensions	1.000	27.4/44.4/100.0	0.599
Mini Nutritional Assessment supplementary	82 active variables	0.906	30.8/44.4/100.0	0.549
All screening instruments supplementary	76 active variables	0.863	31.8/41.2/100.0	0.544
Sex supplementary	83 active variables	0.494	28.6/57.1/95.2	0.526
Residential setting supplementary	83 active variables	0.936	27.4/44.4/100.0	0.599
Sex and setting supplementary	82 active variables	0.604	29.0/45.0/100.0	0.569
Redundant variables pruned	69 active variables	0.664	22.6/48.0/96.3	0.609
Two dimensions retained	84 active variables	0.913	42.1/27.9/100.0	0.596
Ten dimensions retained	84 active variables	0.952	26.2/41.2/100.0	0.626
Refit among women	*n* = 83	0.361	—	—
Refit among men	*n* = 22	0.420	—	—
Refit among community-dwelling participants	*n* = 81	0.826	—	—
Refit among residents of care facilities	*n* = 24	0.430	—	—

Note: The subgroup refits with fewer than 40 individuals are reported for completeness and are not interpretable as corroboration: the number of individuals approaches the number of retained dimensions, and the resulting partition is close to arbitrary. The association with malnutrition risk is preserved under every specification in which it can be computed, with Cramér’s V between 0.526 and 0.626 and all *p*-values below 10^−6^.

**Table A3:** Continuous variables whose separation across clusters exceeds the level generated by the analytical procedure on data with no structure.

Variable	Eta Squared	Adjusted *p*	Exceeds Procedural Null
Protein mass (kg)	0.578	1.1 × 10^−18^	Yes
Fat-free mass (kg)	0.576	1.1 × 10^−18^	Yes
Skeletal muscle mass (kg)	0.576	1.1 × 10^−18^	Yes
Basal metabolic rate	0.576	1.1 × 10^−18^	Yes
Total body water (L)	0.575	1.1 × 10^−18^	Yes
Mineral mass (kg)	0.540	5.2 × 10^−17^	Yes
Average grip strength (kg)	0.489	9.7 × 10^−15^	Yes
Appendicular skeletal muscle mass index (ASM/height^2^)	0.350	1.9 × 10^−9^	Yes
Mini Nutritional Assessment score	0.341	3.3 × 10^−9^	Yes
Timed Up and Go (s)	0.337	4.0 × 10^−9^	Yes

Note: Applying the complete procedure to 200 datasets with all association destroyed by permutation yields a largest eta squared with median 0.184 and 95th percentile 0.273; the ten variables above exceed that percentile. On the same reference, the median number of continuous variables surviving false discovery rate control is 5 (95th percentile 10) against 27 observed (permutation *p* = 0.005). Across the whole family of 84 comparisons, 52 are nominally significant, 50 survive false discovery rate control and 32 survive Bonferroni correction. All *p*-values are Benjamini–Hochberg adjusted across the joint family and are reported as descriptive ordering quantities, not as confirmatory tests.

**Table A4:** Composite nutritional risk index and internal predictive validation.

Quantity	Value
Composite index, range	0–4 positive screens
Distribution (0/1/2/3/4)	26/25/23/22/9
Coefficient alpha across the four instruments	0.587
Mean inter-item correlation	0.265
Composite index by cluster, mean	1.32/1.61/2.48
Eta squared of cluster membership on the composite index	0.137
Two or more positive screens by cluster (%)	41.9/44.4/80.0
Prevalence of malnutrition risk	50 of 105 (47.6%)
Area under the curve, intercept only	0.500
Area under the curve, conventional clinical predictors	0.656 (0.593–0.699)
Area under the curve, cluster membership alone	0.746 (0.721–0.764)
Area under the curve, cluster membership, instrument supplementary	0.704 (0.669–0.726)
Area under the curve, clinical predictors and cluster membership	0.778 (0.735–0.810)
Area under the curve, clinical predictors and cluster membership, instrument supplementary	0.737 (0.690–0.769)
Increment over clinical predictors, non-circular specification	+0.081

Note: Discrimination was assessed by repeated stratified five-fold cross-validation with 50 repetitions; intervals are the 2.5th and 97.5th percentiles across repetitions. Conventional clinical predictors comprise age, sex, body mass index, serum albumin and residential setting. The non-circular specification uses the partition from the sensitivity analysis in which the Mini Nutritional Assessment takes no part in constructing the factor space, and is the basis for the manuscript’s cautious interpretation. Coefficient alpha of 0.587 indicates that the four instruments do not function as parallel indicators of a single construct; the composite is accordingly reported as a count of positive screens rather than as a graded severity measure.

### Appendix A.2. Additional Methodological Details

#### Appendix A.2.1. Formal Specification of the Analysis

The input matrix comprised 105 individuals and 84 active variables grouped into nine thematic domains: nutritional screening (2 variables), sociodemographic characteristics (5), lifestyle (5), comorbidities (7), medication use (11), health assessment (7), risk assessment (5), body composition (11), and blood biomarkers (31). Fifty variables were quantitative and 34 were binary. Binary variables were entered through complete disjunctive coding, with one indicator column per level, so that the coded matrix comprised 118 columns. Groups containing binary variables were declared categorical and handled by the multiple correspondence analysis component of the method. Within those groups, the chi-squared metric was applied, and category coordinates were weighted by the inverse of category frequency so that rare categories could not dominate. No binary variable had fewer than 12 participants in its minority level.

Quantitative variables were centered and scaled to unit variance within their group. Each group was then divided by the square root of the first eigenvalue of its own separate analysis. This is the defining weighting step in Multiple Factor Analysis. It equalizes the maximum axial inertia of each group at unity before the global analysis, preventing any group from dominating the common structure through the number or intercorrelation of its variables. The resulting divisors ranged from 1.155 to 2.639 and are reported for each group in Table A1, together with each group’s contribution to the first two dimensions. Five dimensions, accounting for 38.11% of total inertia, were retained for the clustering step. Distances in this space were Euclidean; agglomeration followed Ward’s criterion in its ward. D2 implementation and the procedure were completed by a k-means consolidation step initialized at the hierarchical centroids.

This weighting scheme is the mechanism by which variable redundancy is handled, which is why no variable selection procedure was applied. Several body-composition measures are algebraically coupled because they derive from a single bioimpedance model. A formal screen identified 34 pairs among the 50 quantitative variables with absolute correlations of at least 0.80, and variance inflation factors exceeding 10^5 for fat-free mass, basal metabolic rate, and total body water. The body composition group absorbed 63.30% of its internal inertia in its own first axis, the highest of any group, and therefore received the heaviest divisor. The visible consequence is that these 11 tightly coupled variables contributed 0.86% to the first common dimension. A data-driven selection on 105 participants would have introduced an additional layer of post-selection inference into an analysis already vulnerable to that criticism. That concern was therefore examined directly in the sensitivity analyses rather than addressed by variable selection in the primary analysis.

All 84 variables were active in the primary analysis, including the Mini Nutritional Assessment score and its derived risk category. No variable was supplementary in the primary specification. The implications of including the Mini Nutritional Assessment in the active set were examined directly in sensitivity analyses and are reported in the Results.

#### Appendix A.2.2. Data Preprocessing

The analytic matrix was complete. All 8820 cells were observed, a condition verified by an assertion in the analysis code that halted execution if it was not met. No imputation was required and none was performed. The 105 participants were those who completed the full assessment protocol. Incomplete assessments were excluded at recruitment rather than carried forward with missing values. This necessarily selects participants able to complete a demanding protocol, which is acknowledged among the study limitations.

A systematic outlier screen identified 31 values lying beyond three interquartile ranges from the nearest quartile. Twenty-eight represented plausible extremes of skewed clinical distributions, mainly C-reactive protein and the leucocyte differential, and were retained because extreme values in inflammatory markers are informative rather than aberrant in a geriatric sample. Three were recording errors and were corrected. Rather than screening against reference ranges, which flags implausible values without recovering the intended ones, the audit used arithmetic identities internal to the full blood count. The identity relating to haematocrit, red cell count, and mean corpuscular volume localized a red blood cell count recorded as 428 M/µL and corrected it to 4.28, indicating a misplaced decimal separator. The identity relating plateletcrit, platelet count, and mean platelet volume localized a mean platelet volume recorded as 91 fL and corrected it to 9.10. The leucocyte differential, which sums to the total count, localized a white blood cell count recorded as 78.5 K/µL and corrected it to 6.18. The consequences were minor. Cluster sizes changed from 61, 18, and 26 to 62, 18, and 25. The adjusted Rand index between the published and corrected partitions was 0.968, with one individual reassigned. The congruence of the first five axes was at least 0.95 throughout, and no conclusion was altered. The correction schedule and the uncorrected matrix are both retained in the deposited package.

Distributional shape was examined for all 50 quantitative variables. After the three corrections, eight were consistent with normality on the Shapiro–Wilk test at a false discovery rate of 5%, and eight had absolute skewness exceeding 2. No transformation was applied. Multiple Factor Analysis operates on the correlation structure of standardized variables and does not assume marginal normality, whereas transforming only a subset of variables would make their loadings non-comparable with those of the untransformed remainder.

Hypothyroidism and hyperthyroidism were recorded as separate indicators because thyroid dysfunction in either direction alters resting energy expenditure, body composition, and appetite through different mechanisms, so collapsing them would obscure rather than simplify interpretation. The recorded prevalence of hyperthyroidism, 23 of 105 participants, warrants explicit caution. It exceeds published estimates for older adults by an order of magnitude; only 2 of those 23 participants receive any thyroid medication, and 2 participants are coded as having both conditions simultaneously, which is not plausible as concurrent diagnoses. By contrast, the hypothyroidism indicator behaves coherently, with 12 of 13 participants receiving levothyroxine. The variable labelled hyperthyroidism therefore probably captures a broader category of self-reported thyroid disorder. The data were not altered pending verification against source records, and the figure should not be interpreted as the prevalence of clinical hyperthyroidism.

#### Appendix A.2.3. Cluster Validation

Four quantitative criteria were reported, with decision thresholds stated in advance. Silhouette widths were computed in the retained factor space, with values below 0.25 conventionally regarded as weak and values above 0.50 as moderate. Bootstrap stability was assessed across 2000 resamples, summarized by the adjusted Rand index against the reference partition and by per-cluster Jaccard coefficients. A cluster below 0.60 was regarded as untrustworthy in isolation and above 0.75 as stable. The between-cluster share of inertia was also reported. Because none of these quantities is interpretable on an absolute scale, the complete pipeline was also refitted on 200 datasets in which every variable was permuted independently, preserving all marginal distributions while destroying association. The resulting reference distributions provide the benchmark against which the observed values are judged. Results are presented in Table 2 and Figure A2.

#### Appendix A.2.4. Sensitivity Analyses

Because the Mini Nutritional Assessment score and its derived risk category were included in the active set in the primary analysis, the finding that one cluster carries high malnutrition risk could partly reflect the analytic specification. The pipeline was therefore refitted with both Mini Nutritional Assessment variables removed from the active set and projected as supplementary variables. The analysis was then repeated with all four screening instruments excluded simultaneously. Agreement with the primary partition and the persistence of the association with malnutrition risk under these specifications are reported in Table 5.

Three further families of sensitivity analyses were performed, as summarized in Table A2 and Figure A3. Sample composition was examined by treating sex, residential setting, and both jointly as supplementary variables, and by refitting the pipeline separately within each sex and each setting. The variable set was examined by constructing a reduced set through iterative removal, from each strongly coupled pair, of the member with the higher mean absolute correlation to the remainder, and refitting the analysis on that set. Dimensionality was examined by repeating the clustering with two through ten retained dimensions of the same factor space, noting that the choice of five was the software default rather than a substantive decision. Subgroup refits with fewer than 40 individuals are reported for completeness but are not interpretable as corroboration, because the number of individuals approaches the number of retained dimensions.

#### Appendix A.2.5. Descriptive Comparisons and Multiplicity

The clusters were constructed by maximizing between-group separation on the same variables subsequently used for their characterization. The null hypothesis of no difference had therefore already been rendered improbable by construction, and a *p* value computed in that setting does not retain its nominal interpretation. All such comparisons are therefore presented as descriptive characteristics. Effect sizes, eta squared for continuous variables and Cramer’s V for categorical variables, carry the main interpretation, and *p* values are retained only as an ordering device. The Benjamini–Hochberg false discovery rate procedure was applied jointly across the full family of 84 comparisons rather than within variable type, because the results are reported together. That criterion was chosen over family-wise error control because the aim is descriptive characterization rather than confirmatory testing, although Bonferroni-adjusted results are also reported for readers preferring the stricter standard.

To quantify the extent of this inflation empirically, the complete procedure from Multiple Factor Analysis through clustering to the testing of every variable was applied to 200 permuted datasets in which no true difference exists by construction. The resulting reference distribution of apparent effect sizes shows how much separation the procedure can generate in the absence of true structure and identifies those observed effects that exceed that benchmark. Results are presented in Table A3 and Figure A5.

#### Appendix A.2.6. Composite Nutritional Risk Index

A composite index was constructed as the count of positive screens across the Mini Nutritional Assessment, the Short Nutritional Assessment Questionnaire, the Council on Nutrition Appetite Questionnaire, and the SARC-F, yielding scores from 0 to 4. Its internal consistency was assessed by coefficient alpha and by the inter-item correlation matrix, in order to determine whether the four instruments behave as parallel indicators of a single construct and whether a simple sum is an appropriate summary. Results are presented in Table A4.

#### Appendix A.2.7. Internal Predictive Validation

Discrimination of malnutrition risk was assessed by repeated stratified five-fold cross-validation with 50 repetitions, summarized by the area under the receiver operating characteristic curve and the Brier score. Four predictor sets were compared: an intercept-only rule, cluster membership alone, conventional clinical predictors comprising age, sex, body mass index, serum albumin, and residential setting, and those clinical predictors together with cluster membership. Because the primary partition was constructed with the Mini Nutritional Assessment variables in the active set, every comparison was repeated using the partition from the sensitivity analysis in which the Mini Nutritional Assessment variables were treated as supplementary. That less circular specification underpins the manuscript’s cautious interpretation. The analysis is internal and estimates performance in new individuals drawn from the same population. It should not be interpreted as external validation. Results are presented in Table A4.

Internal validation suggested an association between cluster membership and malnutrition risk, although this finding should be interpreted cautiously and does not by itself demonstrate clinical utility.

## Appendix B

### Appendix B.1

**Table A5:** Association of categorical and quantitative variables with cluster 1 in hierarchical clustering on principal components (HCPC), *N* = 62.

Category	Cla/Mod (%)	Mod/Cla (%)	Global (%)	*p*-Value	v.test
Female sex	73.49	100.00	79.05	<0.001	6.49
Lack of residential care	72.84	96.72	77.14	<0.001	5.68
Lack of polypharmacy	77.94	86.89	64.76	<0.001	5.58
Lack of malnutrition risk according to MNA	81.82	73.77	52.38	<0.001	5.18
Lack of anticoagulant use	70.73	95.08	78.10	<0.001	4.93
Lack of frailty risk	75.00	83.61	64.76	<0.001	4.71
Higher education	81.63	65.57	46.67	<0.001	4.59
Lack of antidiabetic medication use	67.82	96.72	82.86	<0.001	4.41
Lack of diabetes mellitus	65.56	96.72	85.71	<0.001	3.72
Lack of smoking	65.56	96.72	85.71	<0.001	3.72
Lack of diuretic use	67.90	90.16	77.14	<0.001	3.66
Lack of neurological conditions	67.07	90.16	78.10	0.001	3.44
Lack of heart disease	67.53	85.25	73.33	0.002	3.17
Lack of antihypertensive medication use	77.50	50.82	38.10	0.002	3.15
Lack of hypertension	78.38	47.54	35.24	0.002	3.11
Lack of hyperthyroidism	65.85	88.53	78.10	0.003	2.96
Age below 70 years	70.00	68.85	57.14	0.005	2.81
Lack of GDS risk	67.65	75.41	64.76	0.008	2.63
Lack of hospitalization in the past year	63.64	91.80	83.81	0.012	2.53
Alcohol consumption	66.67	72.13	62.86	0.024	2.27
Hypothyroidism	84.62	18.03	12.38	0.040	2.05
Lack of serious illnesses in the past year	61.96	93.44	87.62	0.041	2.04
Lack of antidepressant use	62.22	91.80	85.71	0.044	2.01
Antidepressant use	33.33	8.20	14.29	0.044	−2.01
Serious illnesses in the past year	30.77	6.56	12.38	0.041	−2.04
Lack of hypothyroidism	54.35	81.97	87.62	0.040	−2.05
Lack of alcohol consumption	43.59	27.87	37.14	0.023	−2.27
Hospitalization in the past year	29.41	8.20	16.19	0.012	−2.53
GDS risk	40.54	24.59	35.24	0.008	−2.63
Age 70 years or above	42.22	31.15	42.86	0.005	−2.81
Hyperthyroidism	30.43	11.48	21.91	0.003	−2.96
Hypertension	47.06	52.46	64.76	0.002	−3.11
Antihypertensive medication use	46.15	49.18	61.91	0.002	−3.15
Heart disease	32.14	14.75	26.67	0.002	−3.17
Neurological Conditions	26.09	9.84	21.91	0.001	−3.44
Diuretic use	25.00	9.84	22.86	<0.001	−3.66
Diabetes mellitus	13.33	3.28	14.29	<0.001	−3.72
Smoking	13.33	3.28	14.29	<0.001	−3.72
Antidiabetic medication use	11.11	3.28	17.14	<0.001	−4.41
Lack of higher education	37.50	34.43	53.33	<0.001	−4.59
Frailty risk	27.03	16.39	35.24	<0.001	−4.71
Anticoagulant use	13.04	4.92	21.91	<0.001	−4.93
Malnutrition risk according to MNA	32.00	26.23	47.62	<0.001	−5.18
Polypharmacy	21.62	13.12	35.24	<0.001	−5.58
Residential care	8.33	3.28	22.86	<0.001	−5.68
Male sex	0.00	0.00	20.95	<0.001	−6.49

Notes: Cla/Mod (%) = percentage of category observations in the cluster; Mod/Cla (%) = percentage of cluster composed of the category; Global (%) = overall prevalence of the category; *p*-values significant at α = 0.05; v.test = z-score indicating strength and direction of association. Binary variables are presented both in presence and absence forms to provide a complete characterization of cluster profiles.

**Table A6:** Association of quantitative variables with cluster 1 in hierarchical clustering on principal components (HCPC).

Variable	v.test	Mean in Category	Overall Mean	SD in Category	Overall SD	*p*-Value
Mini Nutritional Assessment (MNA) score	5.29	25.24	23.82	2.60	3.21	<0.001
Basophils (%)	3.34	0.91	0.81	0.37	0.37	0.001
Low-density lipoprotein cholesterol (LDL, mg/dL)	3.16	129.59	119.21	34.80	39.48	0.002
Total cholesterol (mg/dL)	3.14	223.41	210.89	40.63	47.88	0.002
Non-HDL cholesterol (mg/dL)	2.62	151.57	142.31	36.71	42.36	0.009
High-density lipoprotein cholesterol (HDL, mg/dL)	2.16	71.84	68.60	15.82	17.99	0.031
C-reactive protein (CRP, mg/L)	−2.17	2.42	4.03	1.93	8.89	0.030
Lymphocytes (×10^3^/μL)	−2.31	1.76	2.00	0.43	1.24	0.021
Red cell distribution width (RDW, %)	−2.38	13.54	13.76	0.84	1.13	0.017
Waist-to-hip ratio (WHR)	−3.10	0.89	0.91	0.06	0.07	0.002
Geriatric depression scale (GDS) score	−3.16	5.80	7.20	4.59	5.31	0.002
Eosinophils (×10^3^/μL)	−3.58	0.14	0.18	0.07	0.11	<0.001
Appendicular skeletal muscle index (ASMI)	−3.86	9.12	9.51	0.91	1.21	<0.001
Timed Up and Go test (s)	−4.10	9.41	12.05	3.22	7.73	<0.001
Mineral mass (kg)	−4.23	2.98	3.17	0.32	0.53	<0.001
Skeletal muscle mass (SMM, kg)	−4.36	23.01	24.79	2.69	4.91	0.013
Protein mass (kg)	−4.39	8.29	8.88	0.89	1.62	0.011
Basal metabolic rate (BMR)	−4.47	1285.69	1351.50	97.45	176.94	0.008
Fat-free mass (FFM, kg)	−4.47	42.40	45.44	4.52	8.20	0.008
Total body water (TBW, L)	−4.49	31.12	33.39	3.31	6.06	0.007
Monocytes (×10^3^/μL)	−4.58	0.35	0.42	0.11	0.18	0.005
Neutrophils (×10^3^/μL)	−4.64	3.03	3.52	0.89	1.27	0.004

Note: Binary variables are shown only for the affirmative category, with denominators in the column headers; the complementary category can be derived by subtraction. *p* values are false discovery rate–adjusted across all comparisons and are presented for descriptive purposes only. Full two-level tabulations are available in the analysis package.

**Table A7:** Association of categorical variables with cluster 2 in hierarchical clustering on principal components (HCPC), *N* = 18.

Category	Cla/Mod (%)	Mod/Cla (%)	Global (%)	*p*-Value	v.test
Male sex	77.27	94.44	20.95	<0.001	7.61
Lack of painkiller use	27.91	66.67	40.95	0.019	2.35
Lack of anti-inflammatory use	23.08	83.33	61.90	0.040	2.05
Alcohol use	22.73	83.33	62.86	0.049	1.97
Lack of alcohol use	7.69	16.67	37.14	0.049	−1.97
Anti-inflammatory use	7.50	16.67	38.10	0.040	−2.05
Painkiller use	9.68	33.33	59.05	0.019	−2.35
Female sex	1.20	5.56	79.05	<0.001	−7.61

Notes: Cla/Mod (%) = percentage of category observations in the cluster; Mod/Cla (%) = percentage of cluster composed of the category; Global (%) = overall prevalence of the category; *p*-values significant at α = 0.05; v.test = z-score indicating strength and direction of association. Binary variables are presented both in presence and absence forms to provide a complete characterization of cluster profiles.

**Table A8:** Association of quantitative variables with cluster 2 in hierarchical clustering on principal components (HCPC).

Variable	v.test	Mean in Category	Overall Mean	SD in Category	Overall SD	*p*-Value
Protein mass (kg)	7.75	11.59	8.88	1.17	1.62	<0.001
Skeletal muscle mass (SMM, kg)	7.74	32.98	24.79	3.58	4.91	<0.001
Fat-free mass (FFM, kg)	7.74	59.12	45.44	5.94	8.20	<0.001
Basal metabolic rate (BMR)	7.73	1646.56	1351.50	128.29	176.94	<0.001
Total body water (TBW, L)	7.73	43.48	33.39	4.41	6.06	<0.001
Mineral mass (kg)	7.49	4.03	3.17	0.39	0.53	<0.001
Average grip strength (kg)	6.73	31.01	19.87	7.25	7.68	<0.001
Appendicular skeletal muscle index (ASMI)	6.01	11.08	9.51	0.63	1.21	<0.001
Waist-to-hip ratio (WHR)	4.69	0.98	0.91	0.07	0.07	<0.001
Monocytes (×10^3^/μL)	2.92	0.53	0.42	0.20	0.18	0.004
Neutrophils (×10^3^/μL)	2.69	4.26	3.52	1.27	1.27	0.007
Hemoglobin (HGB, g/dL)	2.65	14.31	13.64	1.13	1.16	0.008
Hematocrit (HCT, %)	2.39	43.63	41.84	3.19	3.48	0.017
Visceral fat level (VFL)	−2.09	11.44	13.80	2.87	5.22	0.036
High-density lipoprotein cholesterol (HDL, mg/dL)	−3.09	56.61	68.60	11.91	17.99	0.002
Percent body fat (PBF, %)	−4.53	29.69	37.70	4.14	8.20	<0.001

Note: Binary variables are shown only for the affirmative category, with denominators in the column headers; the complementary category can be derived by subtraction. *p* values are false discovery rate–adjusted across all comparisons and are presented for descriptive purposes only. Full two-level tabulations are available in the analysis package.

**Table A9:** Association of categorical variables with cluster 3 in hierarchical clustering on principal components (HCPC), *N* = 26.

Category	Cla/Mod (%)	Mod/Cla (%)	Global (%)	*p*-Value	v.test
Malnutrition risk according to MNA	52.00	100.00	47.62	<0.001	6.60
Frailty risk	62.16	88.46	35.24	<0.001	6.46
Residential care	70.83	65.38	22.86	<0.001	5.52
Polypharmacy	56.76	80.77	35.24	<0.001	5.45
Neurological conditions	65.22	57.69	21.90	<0.001	4.70
Diuretic use	62.50	57.69	22.86	<0.001	4.52
Lack of alcohol consumption	48.72	73.08	37.14	<0.001	4.24
Diabetes mellitus	73.33	42.31	14.29	<0.001	4.23
Anticoagulant use	60.87	53.85	21.90	<0.001	4.19
Lack of higher education	39.29	84.62	53.33	0.001	3.73
Anti-inflammatory use	45.00	69.23	38.10	0.001	3.65
Antidiabetic medication use	61.11	42.31	17.14	0.001	3.57
GDS risk	45.95	65.38	35.24	0.001	3.57
Hospitalization in the past year	58.82	38.46	16.19	0.001	3.23
Antidepressant use	60.00	34.62	14.29	0.002	3.09
Smoking	60.00	34.62	14.29	0.002	3.09
Lack of age below 70 years	40.00	69.23	42.86	0.002	3.06
Hypertension	33.82	88.46	64.76	0.003	2.99
Antihypertensive medication use	33.85	84.62	61.90	0.005	2.78
Hyperthyroidism	47.83	42.31	21.90	0.007	2.70
Heart disease	42.86	46.15	26.67	0.014	2.45
Painkiller use	32.26	76.92	59.05	0.034	2.12
Lack of painkiller use	13.95	23.08	40.95	0.034	−2.12
Lack of heart disease	18.18	53.85	73.33	0.014	−2.45
Lack of hyperthyroidism	18.29	57.69	78.10	0.007	−2.70
Lack of antihypertensive medication use	10.00	15.38	38.10	0.005	−2.78
Lack of hypertension	8.11	11.54	35.24	0.003	−2.99
Age below 70 years	13.33	30.77	57.14	0.002	−3.06
Lack of antidepressant use	18.89	65.38	85.71	0.002	−3.09
Lack of smoking	18.89	65.38	85.71	0.002	−3.09
Lack of hospitalization in the past year	18.18	61.54	83.81	0.001	−3.23
Lack of GDS risk	13.24	34.62	64.76	0.001	−3.57
Lack of antidiabetic medication use	17.24	57.69	82.86	0.001	−3.57
Lack of anti-inflammatory use	12.31	30.77	61.90	0.001	−3.65
Higher education	8.16	15.38	46.67	0.001	−3.73
Lack of anticoagulant use	14.63	46.15	78.10	<0.001	−4.19
Lack of diabetes mellitus	16.67	57.69	85.71	<0.001	−4.23
Alcohol consumption	10.61	26.92	62.86	<0.001	−4.24
Lack of diuretic use	13.58	42.31	77.14	<0.001	−4.52
Lack of neurological conditions	13.41	42.31	78.10	<0.001	−4.70
Lack of polypharmacy	7.35	19.23	64.76	<0.001	−5.45
Lack of residential care	11.11	34.62	77.14	<0.001	−5.52
Lack of frailty risk	4.41	11.54	64.76	<0.001	−6.46
Lack of malnutrition risk according to MNA	0.00	0.00	52.38	<0.001	−6.60

Notes: Cla/Mod (%) = percentage of category observations in the cluster; Mod/Cla (%) = percentage of cluster composed of the category; Global (%) = overall prevalence of the category; *p*-values significant at α = 0.05; v.test = z-score indicating strength and direction of association. Binary variables are presented both in presence and absence forms to provide a complete characterization of cluster profiles.

**Table A10:** Association of quantitative variables with cluster 3 in hierarchical clustering on principal components (HCPC).

Variable	v.test	Mean in Category	Overall Mean	SD in Category	Overall SD	*p*-Value
Timed Up and Go test (s)	5.87	19.80	12.05	10.98	7.73	<0.001
Geriatric depression scale (GDS) score	4.91	11.65	7.20	5.07	5.31	<0.001
Visceral fat level (VFL)	3.42	16.85	13.80	6.04	5.22	0.001
Percent body fat (PBF, %)	3.07	42.00	37.70	9.41	8.20	0.002
Neutrophils (×10^3^/μL)	2.95	4.16	3.52	1.49	1.27	0.003
Body fat mass (BFM, kg)	2.72	33.07	28.32	13.14	10.22	0.007
Monocytes (×10^3^/μL)	2.69	0.50	0.42	0.23	0.18	0.007
Red cell distribution width (RDW, %)	2.59	14.26	13.76	1.60	1.13	0.010
Eosinophils (×10^3^/μL)	2.51	0.23	0.18	0.15	0.11	0.012
Body mass index (BMI, kg/m^2^)	2.46	30.86	28.58	7.31	5.42	0.014
Lymphocytes (×10^3^/μL)	2.17	2.46	2.00	2.29	1.24	0.030
Total cholesterol (mg/dL)	−1.96	194.81	210.89	52.98	47.88	0.049
Mean corpuscular hemoglobin concentration (MCHC, g/dL)	−2.12	32.31	32.60	0.85	0.80	0.034
Basophils (%)	−2.27	0.67	0.81	0.35	0.37	0.024
Non-HDL Cholesterol (mg/dL)	−2.33	125.46	142.31	48.62	42.36	0.020
Low-density lipoprotein cholesterol (LDL, mg/dL)	−2.76	100.58	119.21	41.43	39.48	0.006
Hematocrit (HCT, %)	−2.88	40.12	41.84	4.63	3.48	0.004
Hemoglobin (HGB, g/dL)	−3.45	12.96	13.64	1.50	1.16	<0.001
Average grip strength (kg)	−3.93	14.71	19.87	5.70	7.68	<0.001
Mini Nutritional Assessment (MNA) score	−5.85	20.62	23.82	2.26	3.21	<0.001

Note: Binary variables are presented both in presence and absence forms to provide a complete characterization of cluster profiles.

### Appendix B.2

#### Appendix B.2.1. Sensitivity Analyses

Refitting the pipeline with the Mini Nutritional Assessment treated as a supplementary variable, and therefore excluded from axis construction, yielded a partition closely aligned with the primary solution (adjusted Rand index = 0.906), with 102 of 105 individuals retaining their cluster assignment. The association with malnutrition risk remained essentially unchanged: the proportions at risk across clusters were 30.8, 44.4, and 100.0 per cent, compared with 27.4, 44.4, and 100.0 per cent in the primary analysis. Cramer’s V decreased from 0.599 to 0.549, retaining 91.6 per cent of its magnitude (*p* = 1.3 × 10^−7^), and eta squared for the continuous score declined from 0.341 to 0.283. Excluding all four screening instruments simultaneously, leaving 76 active variables, produced a similarly stable partition (adjusted Rand index = 0.863), with the association again preserved (31.8, 41.2, and 100.0 per cent at risk; Cramer’s V = 0.544).

Sex proved to be a principal organizing variable of the factor space. Cluster 1 is composed entirely of women (62 of 62), Cluster 2 almost entirely of men (17 of 18), and Cluster 3 of 20 women among 25 members; the association between sex and cluster membership has a Cramer’s V of 0.846, the largest of any variable in the dataset. Treating sex as supplementary changes the partition appreciably, to an adjusted Rand index of 0.494 with clusters of 63, 21 and 21 members, the lowest agreement observed across all specifications examined. The association with malnutrition risk nonetheless persists under that specification (28.6, 57.1 and 95.2 per cent at risk; Cramer’s V 0.526; *p* = 5.0 × 10^−7^), and under the specification holding out sex and residential setting jointly. Residential setting behaves differently: holding it out leaves the partition almost unchanged (adjusted Rand index 0.936), although it is associated with cluster membership (Cramer’s V 0.638).

Refitting within subgroups gives adjusted Rand indices against the corresponding restriction of the primary partition of 0.826 among the 81 community-dwelling participants, 0.430 among the 24 in residential care, 0.361 among the 83 women and 0.420 among the 22 men. Only the community-dwelling result is interpretable; a nine-group factor analysis of 84 variables on 22 or 24 individuals is not a lower-powered version of the main analysis but a different and largely uninformative exercise, and the three small-subgroup results are reported as a limitation of what this sample can support rather than as corroboration.

Pruning every strongly coupled variable, which removes 15 quantitative variables including all of the algebraically coupled body-composition measures and leaves 69 actives, gives clusters of 53, 25 and 27 members and an adjusted Rand index of 0.664. Agreement is therefore moderate rather than high, and the partition is somewhat sensitive to the redundant block. The substantive finding is not: in the reduced analysis, the proportion at risk across clusters is 22.6, 48.0 and 96.3 per cent, with Cramer’s V of 0.609, marginally higher than in the primary analysis, and eta squared of 0.416. Varying the number of retained dimensions from two to ten gives adjusted Rand indices of at least 0.75 for eight of the nine choices, and the association with malnutrition risk is preserved throughout, with Cramer’s V between 0.596 and 0.626.

#### Appendix B.2.2. Multiplicity and Post-Selection Calibration

Across the joint family of 84 comparisons, 52 are nominally significant at the 5 per cent level, 50 survive Benjamini–Hochberg false discovery rate control, and 32 survive Bonferroni correction. Among the 50 continuous variables, 29 are nominally significant and 27 survive false discovery rate control. These figures are reported for completeness, but the calibration against the procedural null is more informative. Applying the identical procedure to 200 datasets with all associations destroyed by permutation yields a median of 5 continuous variables surviving false discovery rate control, with a 95th percentile of 10, against 27 observed (permutation *p* = 0.005); and a largest eta squared with median 0.184 and 95th percentile 0.273, against 0.578 observed.

Ten continuous variables exceed the 95th percentile of the procedural null: protein mass, fat-free mass, skeletal muscle mass, basal metabolic rate, total body water, mineral mass, average grip strength, the appendicular skeletal muscle mass index, the Mini Nutritional Assessment score and the Timed Up and Go test (Table A3, Figure A5, Appendix A). These are the variables least vulnerable to the objection that post-clustering comparisons are inflated by construction, and the discussion below rests on them. The remaining differences are presented as descriptive characteristics without inferential weight.

#### Appendix B.2.3. The Composite Nutritional Risk Index

The composite index ranges from 0 to 4 positive screens, with 26, 25, 23, 22, and 9 participants at each value. Cronbach’s alpha across the four flags is 0.587, and the mean inter-item correlation is 0.265; individual correlations are heterogeneous, ranging from 0.461 between the Short Nutritional Assessment Questionnaire and the Council on Nutrition Appetite Questionnaire to 0.034 between the Short Nutritional Assessment Questionnaire and the Mini Nutritional Assessment. The four instruments therefore do not behave as parallel indicators of a single latent construct in this sample, so the composite is reported as a count of positive screens rather than as a graded measure of severity. It varies in the expected direction across clusters, with means of 1.32, 1.61, and 2.48 for clusters 1 to 3, respectively (eta squared = 0.137, Kruskal–Wallis *p* < 0.001), and 80.0 per cent of Cluster 3 screening positive on two or more instruments, compared with 41.9 per cent of Cluster 1 (Table A4).

#### Appendix B.2.4. Internal Predictive Validation

Malnutrition risk was present in 50 of 105 participants (47.6 per cent). An intercept-only rule achieves an area under the curve of 0.500 by construction. Conventional clinical predictors achieve 0.656 (95 per cent interval 0.593 to 0.699), and adding cluster membership raises this to 0.778 (0.735 to 0.810). That figure is optimistic, because the partition was constructed with the Mini Nutritional Assessment among the active variables. Repeating the comparison with the partition from the sensitivity analysis, in which the instrument is supplementary, gives 0.704 (0.669 to 0.726) for cluster membership alone and 0.737 (0.690 to 0.769) when added to the clinical predictors, an increment of 0.081 whose interval excludes the clinical model’s point estimate (Table A4). A multidimensional stratification therefore carries information about nutritional risk that conventional clinical variables do not, on a factor space constructed without reference to any screening instrument. This is not evidence of clinical utility, which would require prospective assessment against patient-centred outcomes in a sample not used to construct the stratification.
